# Philadelphia Society of Veterinary Medicine

**Published:** 1892-02

**Authors:** E. S. Muir

**Affiliations:** Secretary, pro. tem.


*Philadelphia Society of Veterinary Medicine.*—Meeting was called to order at 8.15 P. M., with the President, Dr. Ridge, in the chair. The following members were present: Drs. Ridge, Magill, W. L. Zuill, Formad, Harger, Breisacher, Muir, Williams, Maher, J. P. Zuill, Schreiber, Entriken, Pearson, Conrow, Bunting, Eves, Larzelere, Hart, Graham, McCurdy, Fennimore. The first paper read was one by Dr. C. H. Magill on "The Inspection of Veal." He called attention to and described "Monkey Veal," gave a description of what constitutes good veal and compared its appearance and qualities with "Monkey Veal." He said the law as now in force does not protect the public from being imposed upon by the butchers. Dr. S. J. J. Harger then read a very entertaining and instructive paper on "Actinomycosis." He first gave a history of the disease. He stated that the disease is quite common in our cattle, and is sometimes accompanied with tuberculosis. The higher bred animals, he said, were less susceptable to it than the commoner bred ones. He gave the symptoms and general appearance of the parts affected by the disease, also stated that it has been mistaken for contagious pleuro-pneumonia. He said the disease was not confined to cattle alone, but has been noticed in horses, hogs, sheep, and even in the human family. He presented several very fine specimens of sections of heads of cattle affected with the disease, one case in which the sup. maxillary sinus was almost occluded by the growth, also microsopic slides showing the fungus as it appears in colonies and singly. He demonstrated how actinomycosis may be transmitted from one animal to another.

Dr. Magill's paper was then discussed by Drs. Pearson, Schreiber, Williams, Magill, Harger, Formad, Breisacher and Zuill. Considerable interest was shown in this discussion, and many valuable points were brought out.

A very spirited discussion on Dr. Harger's paper was then started by Dr. Schreiber, and participated in by Drs. Harger, Magill, Zuill, Conrow, Williams and others. The point discussed being whether the meat of animals suffering from actinomycosis was fit for food. Dr. Harger said he thought that cases where the disease was localised, to the head, were good for food. Dr. Schreiber took exceptions to this and said any animal that was affected, be it more or less, should be at once condemned and consigned to the refuse vat, as he considered it prejudicial to public health to allow them to be sold for consumption. Further debate on the question was stopped by Dr. Williams, moving that Dr. Formad be requested to write a paper on the disease from a sanitary standpoint, the motion was amended to have the President appoint Drs. Harger and Formad a committee of two to write a paper on the subject from this standpoint. Motion carried as amended. The article in the *Philadelphia Call* of 21st inst., headed "Horses dying in numbers," was then read by the Secretary. A short discussion followed, in which those present stated that the article was unwarranted, as only one case was proven; that of four horses dying in one stable in a short time. Dr. Muir presented a specimen of the larynx of one of the cases. On motion the meeting adjourned, to meet on Tuesday evening, January 19th, 1892.

E. S. Muir, V. M. D.,

*Secretary, pro. tem.*

